# Blue Light Damage and p53: Unravelling the Role of p53 in Oxidative-Stress-Induced Retinal Apoptosis

**DOI:** 10.3390/antiox12122072

**Published:** 2023-12-04

**Authors:** Agnes Fietz, Francesca Corsi, José Hurst, Sven Schnichels

**Affiliations:** 1 Center for Ophthalmology Tübingen, University Eye Hospital Tübingen, 72076 Tübingen, Germany; agnes.fietz@med.uni-tuebingen.de (A.F.); francesca.corsi@phd.unipi.it (F.C.); sven.schnichels@med.uni-tuebingen.de (S.S.); 2 Department of Pharmacy, University of Pisa, 56126 Pisa, Italy

**Keywords:** oxidative stress, AMD, neurodegeneration, cell death, retina

## Abstract

In the digital age, the widespread presence of electronic devices has exposed humans to an exceptional amount of blue light (BL) emitted from screens, LEDs, and other sources. Studies have shown that prolonged exposure to BL could have harmful effects on the visual system and circadian rhythm regulation. BL is known to induce oxidative stress, leading to DNA damage. Emerging research indicates that BL may also induce cell death pathways that involve the tumor-suppressor protein p53. Activated p53 acts as a transcription factor to regulate the expression of genes involved in cell cycle arrest, DNA repair, and apoptosis. This study aimed to explore the implication of p53 in BL-caused retinal damage, shedding light on the potential mechanisms of oxidative-stress-induced retinal diseases. BL-exposed porcine retinal cultures demonstrated increased p53- and caspase-mediated apoptosis, depending on exposure duration. Direct inhibition of p53 via pifithrin α resulted in the prevention of retinal cell death. These findings raise concerns about the long-term consequences of the current daily BL exposure and its potential involvement in various pathological conditions, including oxidative-stress-based retinal diseases like age-related macular degeneration. In addition, this study paves the way for the development of novel therapeutic approaches for oxidative-stress-based retinal diseases.

## 1. Introduction

Elevated oxidative stress levels play a central role in aging processes and cancer, as well as in the development and progression of several neurodegenerative disorders, like Alzheimer’s (AD) and Huntington’s disease [1,2,3]. In ophthalmology, oxidative stress is implicated in the development of eye diseases like senile cataract [4], age-related macular degeneration (AMD) [5], uveitis [6], premature retinopathy [7], and ocular inflammation [8]. AMD is the leading cause of late-age blindness in people over 50. Estimated cases of AMD in 2020 were calculated to be 196 million globally [9]. In Germany alone, 5.8 million people were affected by AMD in 2017, and this number rises every year [10]. Furthermore, for the year 2050, around 77 million cases are estimated in the world to be affected by AMD—compared to 67 million in the year 2015 [11]. Due to the aging population and the lack of treatment options available to combat geographic AMD, it is essential to investigate the underlying mechanisms of this disease and evaluate possible therapeutic approaches. 

Blue light (BL), also known as high-energy visible (HEV) light, has high photochemical energy due to its short wavelengths (400–500 nm). BL damage is mainly caused by excessive production and accumulation of free oxygen radicals (reactive oxygen species, ROS) in mitochondria and photosensitive molecules [12], which causes further damage by overcoming existing protective mechanisms [13,14]. ROS include superoxide anion (O_2_^−^), hydrogen peroxide (H_2_O_2_), nitric oxide (NO), and singlet oxygen (^1^O_2_). Hydroxyl radicals, a highly reactive form of oxygen, can be subsequently generated from H_2_O_2_ [15]. Transmission electron microscopy demonstrated that mitochondria are swollen with disrupted membranes after BL exposure [16]; thus, mitochondria seem to be the main target organelles of ROS [17]. BL exposure could induce and simulate oxidative stress in a more realistic way than with chemical inducers like H_2_O_2_ and thus could be used for investigating the development of many retinal diseases. The ease of application and dosage control of BL offer significant advantages as well. 

ROS causes DNA damage like DNA double-strand breaks [18] and induced atypical gene expression, if the damage is not lethal [19]. This can result in inflammation, reduced autophagy [20], and endoplasmic reticulum stress [16], which is likely to trigger apoptosis. As a component of visible light, BL can penetrate the eye almost unimpeded [21,22]. In the retina, macular yellow pigment with an absorbance spectrum at max. ~460 nm can protect the retina from (photo)oxidative damage [23]; therefore, it can be considered as a BL filter. Evidence suggests that BL exposure may be harmful, particularly in children, where increased screen time has been linked to poorer vision and a higher prevalence of myopia [24]. The causal relationship between short-wave light and myopia is further supported in studies on rhesus monkeys [25]; however, whether BL exposure from screen displays and LEDs can result in retinal damage remains controversial. Almost all studies on BL to date have a common weak point, which is that intense BL exposure is used over a short period of time [26]. However, it is known that BL from yellow phosphor LEDs has a peak emission at ~450 nm +/−35 nm, which is distinct from that of the daylight spectrum [27]. Therefore, persistent sub-threshold damage over time caused by BL is thought to lead to retinal death. Furthermore, excessive BL exposure can promote acute conditions like dry eyes or computer vision syndrome [28]. 

Since 1976, it has been known that BL especially harms rods, primarily through apoptosis [29]. Despite in vitro studies having limitations, as they cannot fully replicate the complexity and context of real biological systems, BL at 435 +/−20 nm can induce apoptosis in retinal cells like retinal pigment epithelium (RPE) cells [30,31]. There is further evidence of BL damage in vivo, albeit only in rodent models [32,33,34]. In AMD, photoreceptors and RPE cells also undergo apoptosis [35]. The role of p53, a tumor-suppressor protein, in BL-induced damage is becoming apparent, particularly in relation to oxidative stress and caspase-induced apoptosis [36], suggesting potential implications for AMD pathogenesis and treatment.

To simulate oxidative diseases such as AMD, we have developed an ex vivo BL damage model that induces oxidative stress and therefore retinal cell damage. To further minimize animal testing, and due to their physiologic similarity to the human eye, organ cultures derived from pig eyes were used for this purpose. Retinal organ cultures were exposed to BL at different durations, and the underlying pathomechanisms were analyzed afterwards. White light (WL) exposure induced significantly less oxidative stress and caspase 3/7 activity compared to pure BL exposure. This is probably because WL also contains red light (RL), which is known to reduce oxidative stress levels in cells and to increase viability [37]. BL exposure resulted in caspase-mediated retinal apoptosis. Treatment with the p53 inhibitor pifithrin α (PFT α) protected against retinal cell death, thus suggesting p53 dependency. The obtained findings pave the way for an ex vivo BL-induced retinal degeneration model via oxidative-stress-induced apoptosis, which allows for animal-free therapy testing in future studies. In addition, we revealed p53 as a potential therapeutic target for retinal degenerative diseases.

## 2. Materials and Methods

### 2.1. Organ Cultures

Porcine eyes were obtained and prepared as described previously [38]. Briefly, they were collected from a local abattoir, which euthanized the pigs by electrocution. The eyes were immediately transported at 4 °C to the laboratory with a maximum time of 3 h after death. The explants were prepared from 6-month-old pigs. Within one hour after the arrival of the eyes at the lab, the pig eyes were cleaned and disinfected, and retinal explants isolated. 

#### Isolation and Cultivation of Retinal Explants

Retinal explants were isolated and kept under culture conditions as described before [39,40]. Briefly, after removing the cornea, lens, and vitreous, a clover-leaf-like structure was generated. Retinas were pierced circularly with a dermal punch (∅ = 3 or 8 mm; Pmf medical AG, Germany). By transferring the eye cup into a petri dish containing neurobasal medium (Neurobasal-A medium; Thermo Fisher Scientific, Karlsruhe, Germany), the retinal explants were carefully removed with a spoon as the retina lifted from the RPE. For histological examinations and the ROS assay, 8 mm retinal explants were placed on a 12-well plate Millicell culture insert (Merck, Germany, with a pore size of 4 μm) containing 100 μL of retina culture medium per insert and 1 ml per well (50 mL Neurobasal-A medium supplemented with 2% B27 (Thermo Fisher Scientific, Karlsruhe, Germany), 1% N2 (Thermo Fisher Scientific, Karlsruhe, Germany), 1% P/S, 0.5 μL CNTF (Merck, Darmstadt, Germany), and 0.5 μL BDNF (Merck, Darmstadt, Germany)) with the ganglion layer (GCL) facing up [40]. Retinal explants were kept under culture conditions at 5% CO_2_ at 37 °C in an incubator for 24 h. For caspase 3/7 and cell viability assays, 3 mm explants were placed and kept under culture conditions in 24-well plate MilliCell culture inserts (Merck, Germany, with a pore size of 4 μm) containing 30 μL of retina culture medium per insert and 500 µL medium per well. Every second day, 80% of the retina culture medium was replaced. Shortly before performing the assays, they were transferred into white 96-well plates (Corning, Kaiserslautern, Germany). 

### 2.2. Light Sources and Exposure Set Ups

#### Blue and White Light Exposure

The 3 and 8 mm retinal explants were exposed from above to ensure normal light exposure. For this purpose, the light path (AquaLight lamp Nr. 117377, Mrutzek Meeresaquaristik GmbH, Ritterhude, Germany) was aligned in a way that one LED illuminated an insert at an intensity of 15 mW/cm^2^ (455 nm). The exposure and following cultivation time varied depending on the experiment and are indicated accordingly in the experiment description. Retinal explants were exposed to WL with an intensity of 15 mW/cm^2^ (AquaLight lamp Nr. 117377, with only WL switched on; Mrutzek Meeresaquaristik GmbH, Ritterhude, Germany). Wavelengths were measured with a Gossen MAVOSPEC BASE analyzer (Gossen, Nürnberg, Germany). Whole pigs’ eyes were exposed to BL in such a way that they could not turn around. To do so, they were placed in a 12-well plate and exposed from above with an intensity of 15 mW/cm^2^ (455 nm). To prevent the cornea from drying, it was ensured that it was moistened with PBS during exposure. Control eyes were in the same room and were also moistened. After exposure, the eyes were placed in a 6-well plate in which 3 mL PBS with 1%P/S was placed. The eyes were placed in the incubator and kept for a further 6 h.

### 2.3. Methods

#### 2.3.1. Ex Vivo Optical Coherence Tomography (OCT)

To mimic the in vivo situation, for which OCT is usually used, a custom-made lens holder was used, as described before [41]. Briefly, an additional lens (78 Diopter lens (78 D double aspheric; Volk optical Inc., Mentor, OH, USA)) was placed in front of the OCT (Spectralis, HRA+OCT device; Heidelberg Engineering, Heidelberg, Germany) to simulate the missing lens and cornea of the eye. Using a custom-made device, the inserts were held in an upright position in front of the OCT. During the measurement, the retinal explants were kept under aseptic conditions. To protect the retina from drying out, each measurement was performed within 5 min. For each retinal explant, three different pictures were quantified with a total of seven, equally distributed, measurements per picture (one in the middle and three equidistant measurements per side). The thickness was determined in pixels. Some 6 h after the generation of the retinal explants, every explant was measured to identify the baseline values. With this value, every later measurement was normalized for each explant. Every measurement was performed with the following parameters: 20 ° line scans, only with sufficient quality (score > 30); and OCT control set to L, ART 100 frames, to reach a similar optical resolution between the explants. 

#### 2.3.2. Quantification of the Retinal Sections (OCT Measurements)

The OCT quantification was performed in a masked fashion using ImageJ (http://rsbweb.nih.gov/ij (accessed on 7 September 2023)). The distances were measured in pixels. The pictures were measured by a second, masked, investigator.

#### 2.3.3. Cell Viability, ROS Assay, and Caspase 3/7 Activity Assay

The viability assay CellTiter Glo 3D (Promega, Walldorf, Germany) was used with 3 mm punches, according to the manufacturer’s protocol. Briefly, explants were transferred into white 96-well plates (Corning, Kaiserslautern, Germany) and mixed with the reagent vigorously at 500 rpm speed on a plate shaker for 5 min. After additional incubation at RT for 30 min, luminescence was measured with a luminometer (Tecan reader SPARK 10M). Promega’s ROS-Glo™ H_2_O_2_ Assay and white 96-well plates (Corning, Kaiserslautern, Germany) were used to determine the level of hydrogen peroxide in the retinal cell cultures. The assay was performed according to the manufacturer’s instructions for the non-lytic version (50 μL from the insert to 50 μL of detection solution). Luminescence was measured with a luminometer (Tecan reader SPARK 10M). To measure caspase 3/7 activity in retinal explants, the caspase 3/7 3D assay (Promega, Walldorf, Germany) was used and the 3 mm punches were again transferred into white 96-well plates and mixed vigorously for 5 min on a plate shaker to ensure a homogeneous solution. After 30 min incubation at RT, luminescence was measured with a luminometer (Tecan reader SPARK 10M). All results were presented in relative light units (RLUs).

#### 2.3.4. Immunostainings

Immunostainings of retinal explants were performed, as described before [42]. Briefly, retinal explants were cryo-protected using Tissue Tek (Sakura, Umkirch, Germany) and frozen in liquid nitrogen. Retinas were cut on a cryostat (12 μm sections) and fixed with ice-cold methanol for 10 min. Afterwards, sections were blocked in 5% BSA/PBS, and the primary antibody was applied overnight at 4 °C (Table 1). Next, sections were washed in TBS-T and a secondary antibody (1:1000) was used for 90 min at RT. After washing and counterstaining with DAPI (4′6′-diamidino-2-phenylindole; Thermo Fisher Scientific, Karlsruhe, Germany), slices were mounted with Fluorsafe (Merck, Darmstadt, Germany). Pictures were taken using a fluorescent microscope (ApoTome2 Zeiss, Germany). 

GFAP expression was quantified by analysis of images with ImageJ (https://imagej.nih.gov/ij/, (accessed on 11 October 2023) National Institute of Health, no copyright protection). Fluorescence intensities for GFAP stainings were measured from the GCL to the outer limiting membrane. Four independent experiments were performed, and intensity values were averaged for at least three images of each experiment, with a total of 12 measurements per condition. To normalize the determined intensity values, the thickness of the inner nuclear layer (INL) was evaluated. For this purpose, the retinal thickness was measured at five specific locations in six images of retinal sections (per treatment). In order to be able to specify this in µM, the scalebar was measured and used to determine the measured length in µM. The mean values were calculated for each image. The GFAP intensities were normalized accordingly.

#### 2.3.5. Western Blot

Retinal explants were collected after indicated amounts of time (as described in each figure legend). For lysing the tissue, 100 µL of cell lysis buffer (Cell Signaling, Danvers, MA, USA) combined with protease inhibitory solution (Merck, Darmstadt, Germany) and PMSF (Merck, Darmstadt, Germany) was used and samples were placed on ice for 30 min. Afterwards, the retinal explants were homogenized by roughly pipetting the samples up and down, followed by centrifugation at 4 °C at maximum speed (13,000 rpm) for 10 min. Supernatant was transferred into new Eppendorf tubes, and a commercial bicinchoninic acid assay (BCA; Thermo Fisher Scientific, Karlsruhe, Germany) was used to determine the protein concentration. Afterwards, 15 µg of each sample was used and 2× Lämmli (Bio-rad, Feldkirchen, Germany) was added for a final concentration of 1×. Some 50% more than was intended to be loaded was prepared. Samples were heated up to 95 °C for 5 min, then cooled down on ice and centrifuged down with a table centrifuge. Then, 10 µL per lane was loaded onto a 10% Mini-PROTEAN TGX Precast Gel (Bio-Rad, Feldkirchen, Germany). Running buffer containing TrisBase (Roth, Karlsruhe, Germany), Glycin (Roth, Karlsruhe, Germany), and SDS (Roth, Karlsruhe, Germany) was used for gel electrophoresis using a PowerPac HC High-current Power Supply (Bio-Rad, Feldkirchen, Germany) with 100 V. Afterwards, transfer was performed at 200 mA for 2,5 h using a nitrocellulose membrane (Cytiva Amersham Protran NC-Membrane, 0.45 µM; Fisher-Scientific, Schwerte, Germany) and Towbin buffer containing TrisBase, Glycine, SDS, and Methanol (Honeywell, Offenbach, Germany). Ponceau staining (ThermoFisher Scientific, Karlsruhe, Germany) confirmed successful transfer. After blocking with Intercept (TBS) blocking buffer (LI-COR, Homburg, Germany) for 30 min, immunostaining was performed using primary antibodies (Table 2) and β-actin as housekeeping for each sample. The next day, membranes were washed with TBS-T, and secondary antibody IRDye 800 RD and IRDye 680 RD (Table 2) were used to visualize protein bands. All antibodies were diluted in Intercept (TBS) blocking buffer containing 0.05% Tween-20. Protein bands were recorded at 700 and 800 nm and evaluated with the Odyssey infrared imager system 2.1 (LI-COR Bioscience, Homburg, Germany).

#### 2.3.6. Inhibition of p53

The functionality of p53 was inhibited using pifithrin α (cyclic pifithrin-⍺-*p*-nitro, ab1460 Abcam, Cambridge, UK) at 1 µM in retinal culture medium for a specific amount of time, as described in the figure legends. Stock solution with 100 µM was aliquoted and frozen at −20 °C. Aliquots were used only once.

#### 2.3.7. Quantitative Real-Time PCR

RNA isolation and cDNA synthesis of porcine retina explants were performed as described previously [43] and according to the manufacturer’s instructions with a MultiMACS cDNA Kit (Miltenyi Biotec, Bisley, UK). To design the primers, Primer3plus software (Version 3.3.0), based on the published GenBank sequence (GenBank: *sus scrofa* taxid:9823) was used (Table 3). The qRT-PCR was performed using a CfX 96 System (BioRad Laboratories, Neuried, Germany) and an SYBR Green SsoAdvancedTM Universal SYBR R Mastermix (Bio-Rad Laboratories, Neuried, Germany). The final primer concentration was 100 nM, with 1 ng/μL of cDNA used in a reaction volume of 20 μL, according to the manufacturer’s instructions. All samples were analyzed in duplicate. The relative expression of the target genes in each group was expressed as the fold changes in gene expression [44]. The expression levels of the target genes were normalized against the housekeeping genes *Actin-β* and *RPL4*.

#### 2.3.8. TUNEL (TdT-Mediated dUTP-Biotin Nick end Labeling) Analysis

Retinal explants or pig eyes were cryo-protected using TissueTek and frozen in liquid nitrogen. Afterwards, the eyes were cut on a cryostat (12 μm sections) and fixed with 4% PFA for 20 min. After washing, sections were incubated in a permeabilization solution (0.1% Triton X-100 (Roth, Karlsruhe, Germany) in 0.1% sodium citrate (Merck, Darmstadt, Germany)). After another washing step, the positive control was treated with DNAse I for 10 min. Controls were washed again and all samples, except the negative control, were incubated with a 50 µL TUNEL reaction mixture as described by the manufacturer (Roche, Mannheim, Germany). Negative controls were treated only with a labelling solution. After one hour at 5% CO_2_ at 37 °C in the incubator, all samples were washed, and nuclei were stained with DAPI for 5 min. Pictures were taken using a fluorescent microscope (ApoTome2 Zeiss, Germany). At least three images from five sections of three independent experiments were analyzed. 

#### 2.3.9. Statistical Analysis

All results are presented as mean ± SEM. Normality was tested with Graphpad PRISM 9 using the Shapiro–Wilk test and Kolmogorov–Smirnov test. In the case of normal distribution, (Welch’s) ANOVA (more than two comparisons) or (Welch’s) *t*-test (two comparisons) were used. Welch’s tests were used when variance and/or sample size between groups differed. Nonparametric tests were used when datasets were not normally distributed (Mann–Whitney test instead of the *t*-test and Kruskal–Wallis instead of ANOVA). Differences were considered to be significant at *p* < 0.05. Statistical differences are indicated as * with *p* < 0.05, ** with *p* < 0.01, *** *p* < 0.001, and **** *p* < 0.0001 compared to the control. Outliers were removed with Graphpad PRISM 9 Identify Outliers-function using the ROUT Method, Q = 1%.

#### 2.3.10. Image Generation

Overview images were generated using the www.biorender.com (Toronto, ON, Canada; accessed on 1 September 2023) platform.

## 3. Results

### 3.1. BL Is the Damaging Component of WL

**Figure 1:**
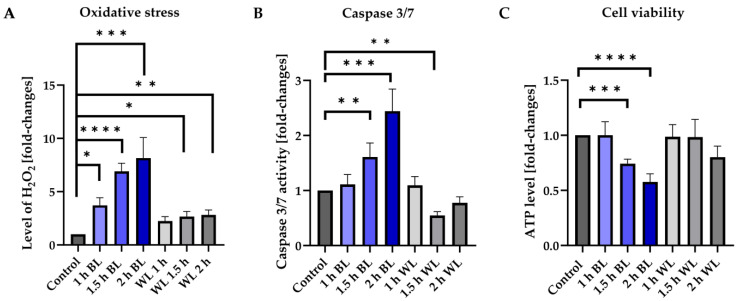
BL is the damaging component in WL and results in retinal cell death. (**A**) Level of hydrogen peroxide (H_2_O_2_) was significantly increased 6 h after blue light (BL) exposure in a dose-dependent manner. White light (WL) exposure, in contrast, did only slightly increase oxidative stress levels. (**B**) BL exposure significantly elevated caspase 3/7 activity in retinal explants 6 h after exposure in a time-dependent manner. No increase was detectable with WL exposures. (**C**) BL-exposed retinal explants showed significantly less cell viability 24 h after exposure. In contrast, WL exposure did not decrease ATP levels. Bar graphs represent the mean with SEM. *n* = 5–10, Welch’s Anova. Control was set to 1. Fold changes are displayed. Statistical differences are indicated as * with *p* < 0.05, ** with *p* < 0.01, *** with *p* < 0.001, and **** with *p* < 0.0001 compared to the control. Abbreviation: h, hours.

Porcine retinal explants were exposed to BL with an intensity of 15 mW/cm^2^ with the GCL facing up, simulating normal light direction. Afterwards, H_2_O_2_ levels, caspase 3/7 activity, and cell viability (ATP levels) were examined. To investigate if single WL exposure has the same effect, retinal explants were exposed for the same durations (Figure 1). Some 6 h after BL exposure, H_2_O_2_ levels were significantly increased depending on exposure duration, with a maximum increase at 2 h of exposure (+8.2 fold ± 4.3) (Figure 1A). WL exposure also significantly increased oxidative stress levels with 1.5 and 2 h of exposure (up to +2.8 fold ± 0.91), but significantly less (*p* < 0.05 for 2 h) compared to BL exposures with the same duration (Figure 1A). Induction of caspase 3/7 activity 6 h after exposure was only detectable for BL exposures, and not for (single) WL exposures (Figure 1B). The increase in caspase 3/7 activity was again dependent on the exposure time, with 2 h of BL resulting in a +2.4 fold ± 0.98 increase (*p* < 0.001) compared to the unexposed control (Figure 1B). Some 24 h after BL or WL exposure, ATP levels were determined to measure retinal viability (Figure 1C). Consistent with the previous measurements, the decrease of ATP after BL exposure was again dependent on the duration time (Figure 1C), with the lowest value for 2 h of BL exposure (−43%, *p* < 0.0001). In contrast, no significant decrease of ATP levels, thus viability, was detectable after WL exposure (Figure 1C). Therefore, BL appears to be the harmful component in WL, and single WL exposure does increase oxidative stress slightly, but not enough to induce cell death. 

### 3.2. Müller Cells React to BL Exposure

**Figure 2:**
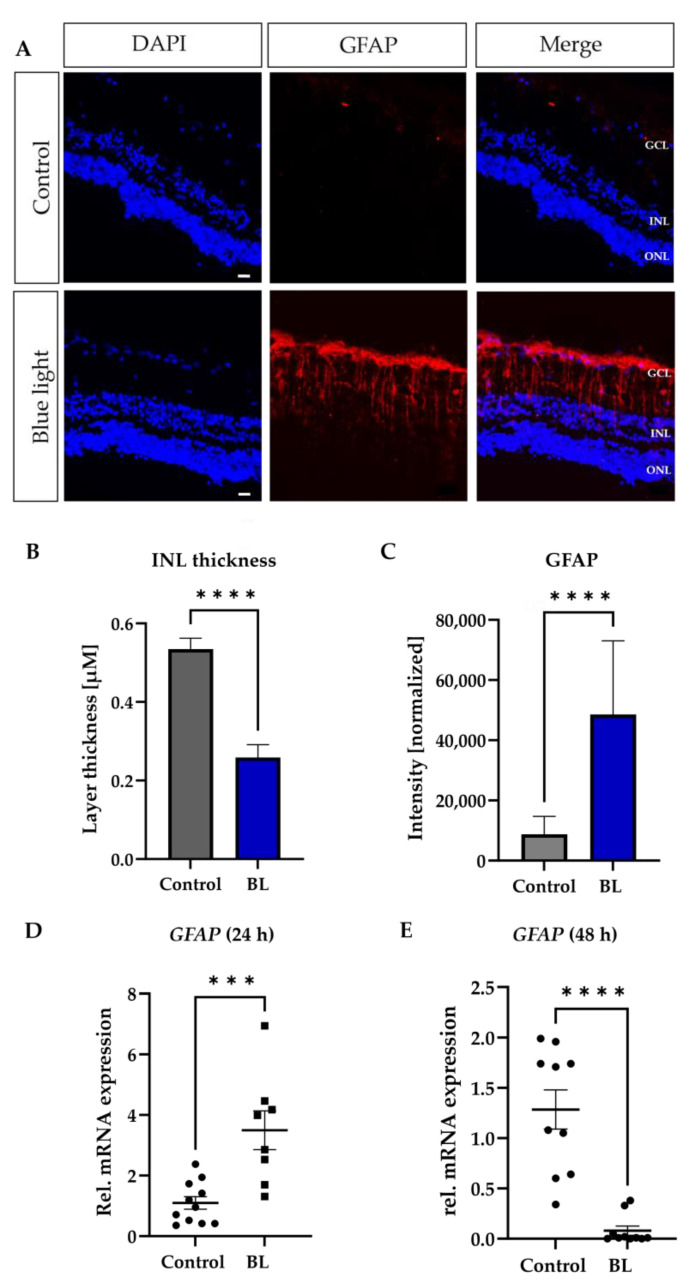
BL induced gliosis and resulted in cell death of Müller cells over time. Retinal explants were exposed to blue light (BL) for 1.5 h and further kept under culture conditions for 24 h. (**A**) Significantly increased GFAP expression was detectable due to BL exposure. Scale bar 50 µM. (**B**) The thickness of the inner nuclear layer (INL) was significantly reduced due to BL exposure. (**C**) GFAP intensity was significantly increased in retinal explants due to BL exposure. GFAP signal was normalized to INL thickness. (**D**) mRNA expression of *GFAP* was induced due to BL exposure in the retinal explants 24 h after exposure, but (**E**) significantly reduced after 48 h, probably reflecting cell death. All data are presented as mean ± SEM. Welch’s students *t*-test. (**A**,**C**): *n* = 6; (**D**,**E**): *n* = 12. Statistical differences are indicated as *** with *p* < 0.001, and **** with *p* < 0.0001 compared to the control. Abbreviations: GCL, ganglion cell layer; INL, inner nuclear layer; ONL, outer nuclear layer; h, hours.

Retinal explants allow the analysis of different cell types via specific markers. Since Müller cells (MCs) are surprisingly sensitive to BL exposure [38], and thought to play a role in oxidative stress-driven AMD progression, the effect of BL especially on this cell type was investigated in the next step. BL exposure (1.5 h) resulted in elevated GFAP expression in the GCL and INL after 24 h (Figure 2A), representing reactivity (gliosis) of the MCs. Also, BL resulted in a significantly reduced thickness of the INL layer (−49%, *p* < 0.0001) containing MC nuclei, probably due to cell death (Figure 2B). Quantification of the fluorescence signal confirmed the previous GFAP finding (Figure 2C, +200%, *p* < 0.0001), as well as enhanced *GFAP* gene expression (Figure 2D). Some 48 h after exposure, a significant decrease (−38%, *p* < 0.0001) of *GFAP* expression was also detectable at the gene level (Figure 2E).

### 3.3. BL Damages Retinal Cells and Leads to Accumulation of Rhodopsin

**Figure 3:**
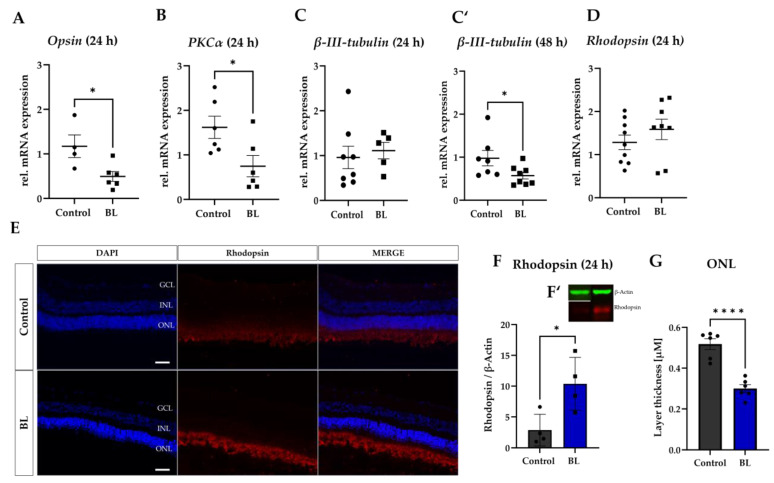
Blue light decreased *opsin*, *PKC-α,* and *β-III-tubulin* expression but increased rhodopsin expression in retina explants. Retinal explants were exposed to blue light (BL) for 1.5 h and further incubated under culture conditions for the indicated time frames. (**A**,**B**) Significantly decreased *opsin* and *PKC-α* mRNA levels were detectable in BL-exposed retinal explants. (**C**,**C′**) *β-III-tubulin* expression was not changed after 24 h but significantly decreased 48 h after BL exposure. (**D**,**E**) Rhodopsin protein levels were significantly increased 24 h after BL exposure in retinal explants, as demonstrated by immunohistology (**E**) and Western blot analysis (**F**). (**F**) A significant increase in rhodopsin protein levels was detectable after 24 h. (**F′**) Rhodopsin expression was normalized to the expression of the housekeeping protein β-Actin. Representative cropped blot images are shown. (**G**) The thickness of the outer nuclear layer (ONL), which contains the nuclei of photoreceptors, was significantly reduced 24 h after BL exposure (*n* = 6). All data are presented as mean ± SEM. Welch’s students *t*-test. *n* = 4–8. Statistical differences are indicated as * with *p* < 0.05 and **** with *p* < 0.0001 compared to the control. Scale bar: 50 µM. Abbreviations: GCL, ganglion cell layer; INL, inner nuclear layer; ONL, outer nuclear layer; h, hours.

The expression of further cell type markers was analyzed in the next step. To this end, cones (*opsin*), rods (*rhodopsin*), retinal ganglion cells (*β-III-tubulin*), and retinal bipolar cells (*PKC-α*) were investigated (Figure 3A–D).

Although the mRNA expression of *opsin* and *PKC-α* was already significantly reduced 24 h after BL exposure (−33%, *p* < 0.05; −19%, *p* < 0.05) (Figure 3A,B), the *β-III-tubulin* expression did not decrease before 48 h of subsequent cultivation (Figure 3C,C′). In contrast to the reduction of these retinal markers, rhodopsin expression significantly increased at the protein level (+3.3 fold ± 2.8, *p* < 0.05) and slightly at gene level after 24 h (Figure 3D–F). However, the thickness of the ONL was reduced 24 h after BL exposure (Figure 3G). Therefore, BL exposure led to accumulation of rhodopsin in the retinal explants, which could interfere with the cellular process in the photoreceptors and increase the oxidative stress levels. This in turn could result in photoreceptor cell death.

### 3.4. Apoptosis Induced by Blue Light Depends on the Exposure Duration

**Figure 4:**
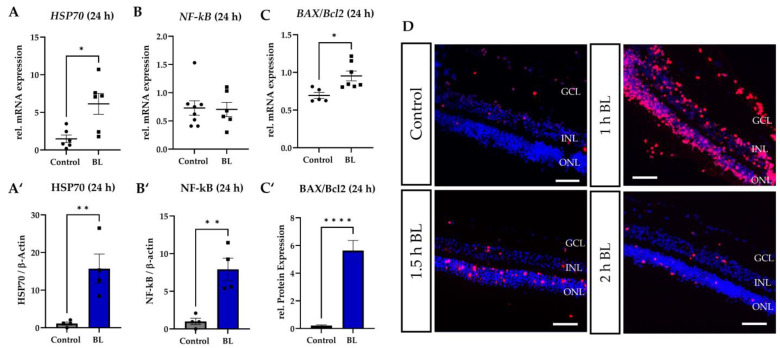
Blue-light-induced oxidative-stress-driven apoptosis in retinal explants. (**A**–**C**) Retinal explants were exposed to blue light (BL) for 1.5 h and further kept under culture conditions for 24 h. (**A**,**A′**) Significantly increased *HSP70* mRNA levels as well as protein levels were detectable in the BL-exposed retinal explant. (**B**,**B′**) BL exposure resulted in no increase in *NF-kB* mRNA expression, but in significantly elevated NF-kB protein expression. (**C**,**C′**) The BAX/BCL-2 ratio was significantly increased at mRNA as well as at the protein level due to BL exposure, compared to the control. (**D**) TUNEL assay was performed 24 h after BL exposure to determine apoptotic cells (red staining). Different exposure durations were investigated with 1 h of exposure resulting in the highest amount of apoptotic (TUNEL+) cells, probably because retinal cells died earlier in longer exposure durations. Representative pictures are shown (*n* = 4). Scale bar 50 µM. All data are presented as mean ± SEM. Welch’s students *t*-test. qRT-PCR: *n* = 5–7. Statistical differences are indicated as * with *p* < 0.05, ** with *p* < 0.01, and **** with *p* < 0.0001 compared to the control. Abbreviations: GCL, ganglion cell layer; INL, inner nuclear layer; ONL, outer nuclear layer; h, hours.

To further analyze the observed loss of retinal cells after BL exposure, stress and apoptosis markers were evaluated (Figure 4). The strong increase in heat shock protein 70 (HSP70) 24 h after 1.5 h BL exposure (+4.2 fold ± 3.4, *p* < 0.05 at mRNA level and +14.2 fold ± 7.7, *p* < 0.01 at protein level) demonstrated the upregulation of the oxidative stress defense in the retinal explant due to increased ROS (Figure 4A,A′). Multiplayer nuclear factor kappa B (NF-kB), which is involved in stress responses and inflammation, was also upregulated at the protein level 24 h after exposure (+7.97 fold ± 2.9, *p* < 0.01) (Figure 4B,B′). Some 48 h after exposure, *Nf-κB* expression was also increased at the gene level (+2.85 fold ± 2.2, *p* < 0.05) (Figure S1C). In this context, a distinct upregulation of pro-inflammatory and cell death inducing cytokine *TNF-α* was detectable after 24 h (Figure S1A), but not after 48 h (Figure S1B), demonstrating the time-dependent regulation of cell death and/or inflammation. More specific, pro-apoptotic BAX expression was evaluated compared to its inhibitor, thus anti-apoptotic, BCL-2 expression 24 h after BL exposure (Figure 4C,C′). At mRNA (+1.44 fold ± 0.127, *p* < 0.05) and protein level (+28.3 fold ± 0.73, *p* < 0.0001), a significant increase in free BAX was detectable (Figure 4C,C′), indicating induction of apoptosis due to BL exposure.

Apoptosis was further confirmed via TUNEL assay, in which apoptotic cells are visualized in red (TUNEL+ cells) (Figure 4D). Some 24 h after exposure, 1 h of BL resulted in the highest number of TUNEL+ cells, compared to 1.5 h and 2 h of exposure (Figure 4D), probably because longer durations resulted in cell death earlier and the dead cells were already lost after 24 h. This was also underlined with more TUNEL+ cells 6 h after 2 h BL exposure, compared to 1 h of BL exposure (Figure S1D). Therefore, longer BL exposures resulted in apoptosis earlier, compared to shorter exposures.

In this context, we also investigated whether irradiation with BL can also lead to apoptosis in a whole eye. Whole pig eyes were therefore irradiated with BL for 3 h and placed in PBS for a further 6 h in the incubator (Figure S2A). It was found that irradiation of a whole eye also led to an increase in TUNEL+ cells (Figure S2B) and therefore to a significant induction of apoptosis (Figure S2C). A large proportion of the TUNEL+ cells counted were in the photoreceptor layer (ONL) (Figure S2C).

### 3.5. BL Induced the Expression of Modified p53

**Figure 5:**
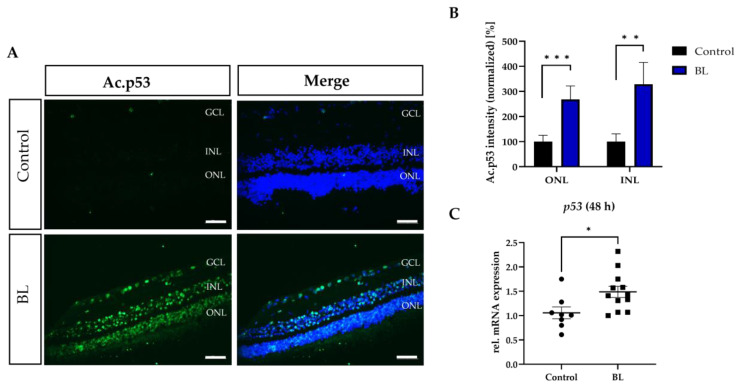
Blue light exposure increased p53 expression in retinal explants. Retinal explants were exposed to blue light (BL) for 1.5 h and further kept under culture conditions for 24 h. (**A**,**B**) Fluorescence intensity for acetylated (lysine 382) and thus activated p53 was significantly upregulated in BL exposed retinal explants. Data were normalized to the overall DAPI fluorescence signal. *n* = 3 with 3 sections each. Control was set to 100%. Scale bar 50 µM. (**C**) p53 gene expression was upregulated 48 h after BL exposure. Welch’s students *t*-test. Statistical differences are indicated as * with *p* < 0.05, ** with *p* < 0.01, and *** *p* < 0.001 compared to the control. Abbreviations: Ac, acetylated; GCL, ganglion cell layer; INL, inner nuclear layer; ONL, outer nuclear layer.

To specifically verify apoptosis as induced cell death, the expression of p53 was investigated in the BL damage model. Since p53 unfolds its functionality as an apoptosis inducer not only through accumulation or increased protein expression, but also through various post-translational modifications, acetylation at lysine residue 382 was investigated. This modification is important to decrease the ubiquitination of p53 via its inhibitor E3 ubiquitin-protein ligase MDM2, thus increasing its half-life [45]. Furthermore, this acetylation is required for BAX activation, generation of ROS, and induction of apoptosis [46]. In BL-exposed retinal explants, more acetylated-p53-positive cells were detected compared to controls (Figure 5A). 

A more detailed analysis of the signal intensity revealed significantly more acetylated p53 expression due to BL exposure (INL: +228%, *p* < 0.01; ONL: +168%, *p* < 0.001) (Figure 5B). Interestingly, significantly more acetylated p53 was found in the INL, compared to the ONL (*p* < 0.01) (Figure 5B). A significant increase in gene expression was seen after 48 h (+1.41 fold ± 0.17, *p* < 0.05) (Figure 5C). Besides the well-known increase in p53 protein levels to stabilize p53 and activate its pro-apoptotic functions, an increased translation of p53 is described as another critical step after irradiation [47]. Therefore, increased mRNA levels of p53 by BL exposure can lead to the accumulation of p53 protein and result in increased apoptosis.

### 3.6. BL-Induced Retinal Damage Can Be Reduced by p53 Inhibition

**Figure 6:**
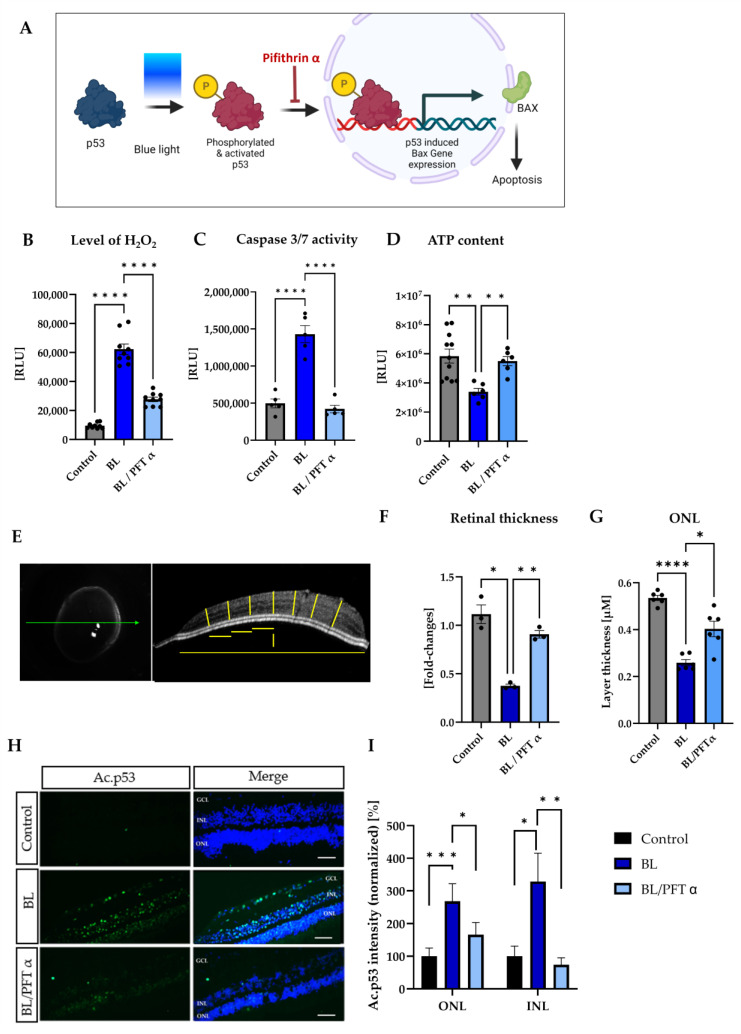
Inhibition of p53 by PFT α rescues BL-damaged retinal explants. To analyze p53 dependency, a transcriptional inhibitor of p53 was used. (**A**) Pifithrin α (PFT α) blocks post-translational modifications of p53 and thus the expression of its downstream targets and induction of apoptosis. Treatment with one µM PFT α significantly reduced the BL-induced (**B**) oxidative stress levels and (**C**) caspase 3/7 activity 6 h after 1.5 h of BL exposure. (**D**) ATP levels, reflecting the viability of the cells, were significantly reduced after 24 h due to BL exposure but increased again after PFT α treatment. (**E**) Representative OCT picture with measurements indicated as yellow lines. After determining the middle, the thickness of the section was measured. From the middle, three equidistant measurements were generated per side, resulting in a total of seven measurements per picture. For each retina, three different pictures were quantified, and the explant was always imaged in the same direction. (**F**) Retinal thickness significantly decreased 24 h after BL exposure but increased again with PFT α treatment. *n* = 4. (**G**) Evaluation of the outer nuclear layer (ONL) thickness demonstrated significant reduction due to BL and increased thickness in retinal explants treated with PFT α after exposure. *n* = 6. (**H**,**I**) Acetylated p53 expression was analyzed 24 h after BL exposure. BL significantly increased the expression in the ONL and INL layer, but inhibition of p53 decreased the amount of acetylated p53 again. *n* = 3 with 3 sections each. Control was set to 100%. Scale bar 50 µM. Welch’s ANOVA. Statistical differences are indicated as * with *p* < 0.05, ** with *p* < 0.01, *** with *p* < 0.001, and **** with *p* < 0.0001. Abbreviations: Ac, acetylated; RLU, relative light units; GCL, ganglion cell layer; INL, inner nuclear layer; ONL, outer nuclear layer.

To further investigate the role of p53 in BL-induced retinal cell death, the p53-inhibitor, pifithrin-α (PFT-α), was used in the next step. PFT-α is a widely used specific inhibitor of p53-transcription activity (Figure 6A). It resembles a small, cell-permeable molecule that binds to p53 and affects its post-translational modifications without changing the total protein level [48]. Treatment of retinal explants with 1 µM PFT-α resulted in a significant decrease in oxidative stress levels (−56%, *p* < 0.0001) (Figure 6B) and caspase 3/7 activity (−71%, *p* < 0.0001 (Figure 6C), compared to BL-exposed retinal explants. Also, ATP levels, which were significantly reduced by BL exposure, were again markedly increased by p53 inhibition (+168%, *p* < 0.01), compared to BL-exposed explants (Figure 6D), indicating improved viability of the exposed retinal explants. To further investigate retinal survival, the thickness of the retinal explants was measured by optical coherence tomography (OCT) (Figure 6E). A significant reduction in retinal thickness 24 h after BL exposure (−67%, *p* < 0.05) (Figure 6F) was detectable. Compared with the exposed explants, retinal thickness increased significantly with PFT α treatment (+141%, *p* < 0.01), corresponding to increased cell survival by p53 inhibition. Taken together, these data demonstrate that B-, and therefore oxidative stress-, induced apoptosis, is mediated by p53 (Figure 6). Because PFT α affects post-translational modifications of p53, acetylation at lysine residue 382 was reexamined (Figure 6G). A strong reduction in the expression of acetylated p53 after treatment with PFT α on BL-exposed explants was detectable (Figure 6G). Quantification of the signal intensity revealed a strong effect in the INL, where the nuclei of MCs are located, with a decrease of 78% (*p* < 0.01) by PFT α treatment (Figure 6H). However, a reduction of the signal intensity and therefore a decreased expression was detected in the ONL, where the photoreceptors are situated (−40%, *p* < 0.05) (Figure 6H). Therefore, it can be concluded that binding of PFT α to p53 prevents acetylation at Lys382, resulting in decreased apoptosis induction.

To verify whether PFT α supports the survival of MCs in particular, the activation of MCs was analyzed by GFAP staining (Figure S23) and the thickness of the INL was examined (Figure S2B). The increased cell survival was investigated by the number of nuclei (DAPI intensity) in the INL of BL-irradiated explants (Figure S3B). Here, treatment with PFT α also led to an increase of 36% (*p* < 0.05) (Figure S3B), compared to BL-exposed explants. A significant reduction in GFAP expression by PFT α treatment after BL exposure (−46%, *p* < 0.0001) (Figure S3A,C) was also detectable. Therefore, it can be assumed at this point that cell survival of MCs is supported by p53 inhibition in the BL damage model.

## 4. Discussion

Many in vitro studies present supporting evidence for BL-induced retinal damage. However, due to the intrinsic limitations of these studies in simulating complexity, they do not do justice to complex interactions in the eye [49,50,51]. Nevertheless, it has been shown that BL at 435 +/−20 nm can induce apoptosis in retinal cells through the activation of caspase 3 [12,52,53]. In vivo, studies on mouse or rat models also demonstrated damage caused by BL [28,53,54,55], but the eyes of these rodent models only resemble human eyes to a limited extent. For example, rodents lack an area of high visual acuity, like the macula, which is essential for the investigation of AMD. In contrast, the porcine retina resembles the human morphologically and physiologically better than rodent eyes [56] and possess a “visual streak” which is also an area of higher visual acuity [57]. Current data on BL damage indicate that the degree of damage is related to factors such as the intensity of BL received, distance of illumination, and spectrum of the light source.

We were able to proof that BL exposure increased H_2_O_2_ levels and caspase 3/7 activity in porcine retinal explants in a dose-dependent manner (Figure 1A,B). Likewise, a decreased cell viability was observed, demonstrating that cell death is induced by BL exposure (Figure 1C). The expression of HSP70, which is among the first candidates responding to oxidative stress to protect the cells, is also significantly increased due to BL damage (Figure 4A). We also demonstrated that BL exposure results in apoptosis in whole pig eyes (Figure S2), where the cornea and the vitreous are included. Since AMD and AD are both late-onset, neurodegenerative diseases that share several clinical and pathological features, such as the presence of oxidative stress and inflammation [58], and cellular aging displays similar features in the retina and brain tissues, research on AMD could also provide a deeper understanding of AD, and vice versa. The observed induction of oxidative stress-mediated cell death due to BL exposure could therefore also provide a platform for investigating the pathways leading to AD onset and progression.

The tumor-suppressor protein p53, a key player in apoptosis induction, has not yet been revealed to have a direct role during BL exposure, although it is well known that high levels of oxidative stress lead to its activation [36]. Previously, our lab showed a surprising sensitivity of retinal Müller cells (MCs) to BL, and therefore to oxidative stress, which finally resulted in p53-mediated apoptosis, thus giving an initial indication for a direct role of p53 in BL-induced retinal damage [38]. In the eye, p53 is associated with retinal responses to radiation [59,60], oxidative stress, ischemia, and the development of retinoblastoma [61,62,63]. Miller et al. further described the role of p53 in caspase-induced apoptosis in the 661W photoreceptor cell line after chemically induced oxidative stress [60], and BL induced caspase-mediated apoptosis in these cells [52]. In RPE cells, elevated oxidative stress levels resulted in p53 activation [62]. The studies of Bhattacharya et al. suggest that age-related post-translational modifications of p53 limit its binding to its inhibitor MDM2, thereby activating caspase 3, inducing apoptosis, and thus triggering the pathogenesis that could lead to AMD. Moreover, components of the p53/MDM2 signaling pathway have been identified which could serve as targets for the treatment of the disease [64]. Recent data have shown that exposure to BL can induce apoptosis, suggesting that p53 may play a role in BL-induced damage. 

Grimm et al. discovered that mice lacking rhodopsin are protected against BL damage, thus BL damage is rhodopsin mediated [65]. Rhodopsin resembles the light receptor in rod outer segment disc membranes, which are important for phototransduction and, thus, vision. BL can cause translocalization of rhodopsin from the inner and outer segments to the ONL, resulting in apoptosis. The regeneration of rhodopsin resembles a critical step in the visual cycle because it enables photoreceptor cells to respond to light stimuli. However, regeneration could lead to oxidative stress. For example, during phototransduction, the energy transfer associated with photon absorption may generate ROS as a byproduct. Another step in the visual cycle is the change in membrane potential, which results in activation of the NADPH oxidase, which naturally generates ROS as a byproduct of its activity. Rhodopsin can accumulate in the retina due to dysfunction of the visual cycle, leading to the degeneration of photoreceptor cells. In this context, Grimm et al. demonstrated that BL can restore activatable rhodopsin and thus can increase its photon absorption capacity [65]. Thus, the observed accumulation of rhodopsin after BL exposure (Figure 3) may be a result of greater regeneration and could increase oxidative stress and cellular damage by, e.g., blocking intracellular traffic. Although opsins are not a primary focus in AMD pathogenesis, dysfunction in the visual cycle could potentially impact retinal health and thus contribute to retinal degeneration. Therefore, the accumulation of rhodopsin in rod photoreceptor cells due to BL exposure (Figure 3) could increase oxidative stress levels, resulting in cell death and inflammation, further contributing to the progression of AMD. This would also explain the great amount of apoptotic cells in the ONL of BL exposed whole pig eyes (Figure S2).

Inner retinal cells like MCs, in contrast to RPE cells, lack photosensitizers like melanin, which make the cells vulnerable to photochemical damage [66,67]. They resemble the most predominant cell type of vertebrate retinas and are considered to act as optical fibers both in vitro and in vivo [68]. MCs leak BL to the surrounding rods [69], meaning that mainly rod photoreceptors are affected by BL exposure. In this context, it is also possible that they leak toxic products to rod photoreceptors [70]. Under stress conditions, increased GFAP expression occurs in MCs, resulting in degenerative alteration of the inner retina [33]. This points out a possible and important role for MCs in BL-induced retinal degeneration. In a previous publication, we demonstrated the sensitivity of these cells towards BL exposure [38]. In BL-exposed retinal explants, a significant increase of *GFAP* mRNA expression 24 h after exposure was measured (Figure 2D), in correlation to our histological examinations revealing a heavily increased protein level of GFAP (Figure 2A,C). This upregulation might also explain the loss of cells after 24 h in the INL of BL-exposed retinal explants (Figure 2B). In accordance, GFAP expression decreased 48 h after BL exposure (Figure 2E). MCs are known to recycle chromophores and supply them selectively back to cones [71]. The activation of MCs due to BL exposure, followed by their cell death (Figure 2), could reduce their ability to regenerate cone opsin, and therefore be a cause of the decreased *Opsin* expression, which was found 24 h after BL exposure (Figure 3). Also, during AMD development, it is known that after the dysfunction of RPE cells, rod loss occurs followed by the degeneration of cones [72]. Thus, as cone photoreceptors degenerate, the number of cells containing opsin decreases, leading to a reduction in the levels of opsins in the affected areas of the retina. Therefore, decreased *Opsin* expression due to BL exposure could also be a result of cone cell death. BL exposure further led to decreased *PKC-α* and *β-II-tubulin* expression (Figure 3B,C′), representing the degeneration of retinal bipolar and ganglion cells.

The induced expression of *TNF-α* mRNA by BL in retinal explants (Figure S1A) indicates both immune system activation [73] as well as a possible initiation of the extrinsic apoptosis pathway [74]. During the development of AMD, there is a release of various inflammatory cytokines [75], including TNF-*α*, which is why the expression induced by BL is consistent with AMD development. Evidence that BL in particular induces apoptosis was found with elevated caspase 3/7 activity (Figure 1B), free BAX (Figure 4C), and more TUNEL+ cells (depending on exposure duration and cultivation time) (Figure 4D and Figure S1). Another marker of apoptosis is p53 [76]. The expression of acetylated p53 (Lys-382) was significantly increased in BL-exposed retinal explants (Figure 5A,B), indicating a role of p53 in BL-induced cell death. Under normal conditions, the p53 protein is degraded very fast [76,77], but protein levels can remain elevated due to posttranslational modifications triggered by various types of stress. The lysine 382 acetylation site lies within the MDM2 binding region [75,78]; thus, acetylation leads to reduced MDM2-triggered degradation, maintaining p53 at a high level [79]. In order to further demonstrate that p53 plays a role in BL-induced retinal degeneration, an inhibitor of p53, PFT-α, was used. A single treatment with 1 µM PFT-α immediately after BL exposure resulted in a significant reduction of oxidative stress levels (Figure 6B) as well as caspase 3/7 activity (Figure 6C). As the elevated expression of acetylated p53 due to BL exposure was decreased by PFT-α, the treatment likely blocked p53 post-translational modifications as well. Inhibition of p53 also affected MC survival, by decreasing GFAP expression of BL-exposed retinal explants (Figure S2). Therefore, the BL-induced retinal apoptosis seems to be p53-mediated.

By contrast, a potential role of p53 was not found in WL-induced photoreceptor cell death in mice in 1998 [80]. 

The amount of light considered to be damaging to the human eye depends on several factors, such as the duration of exposure, the specific wavelengths used, and the age of the eye. Miller et al. did not quantify any data shown; thus, evaluation of the representative pictures remains subjective, and the exposure modality is also not comparable with the exposure applied in our study. There is also a lack of knowledge about the proportions of blue light and RL in their WL exposure, and their model system—mouse—has major differences from the pig ex vivo model we use, which is in general more similar to the human eye. Also, the impact of p53 in the light-induced damage model might be limited to specific cell types, like MCs, and affect other cell types, e.g., photoreceptors, only in a slight manner. With a single WL exposure, we detected only a slight increase in oxidative stress levels, not resulting in caspase 3/7 activity in the retinal explant (Figure 1A,B), which stands in contrast to the damaging effect of a pure, single BL exposure (Figure 1). This confirms our hypothesis that BL exposure, but not single-time WL exposures, especially causes p53-mediated apoptosis. WL contains different wavelengths in different amounts including BL but also RL, which reduces oxidative stress levels and displays a neuroprotective function [37], and thus it may result in a neutralization of the damaging BL effect. This agrees with safety studies, showing that the retina is more susceptible to BL around 460 nm than to RL around 630 nm [81] and the finding that RL decreases apoptosis in BL-exposed ARPE-19 cells [82]. Therefore, it also can be expected that RL in WL-emitting LEDs minimizes the p53-mediated apoptotic induction by BL, depending on the ratio to each other. It is important to note that the human eye has developed techniques to handle a wide range of light intensities and wavelengths, including BL, without causing immediate harm under normal conditions.

There is an ongoing debate about how BL damage can occur in everyday life. BL accounts for 25% of the sun’s rays, ~30% of radiation emitted by electronic devices, and up to 40% emitted by indoor lights. However, these BL exposures have different intensities (i.e., BL hazard in sunlight exposures ~18–25 Wm^−2^ in contrast to 0.230 Wm^−2^ in electronical devices [83]) and the exposure times also vary greatly. While the sun is only looked at directly for a very short time, monitors/televisions/cell phones expose eyes for many hours. During this time, the eye is rarely moved and blinking is also reduced. American teenagers spend more than 8 h a day on digital devices, and it is almost 6 h for children between 8 and 12 years old [84]. The BL of white LEDs was shown before to cause retinal toxicity at household exposure levels. However, these studies were carried out in the short term [85]. Comparing the spectral emissions of light from sunlight and LED sources, there are clear differences—a cool white LED has a spike of luminescence in the BL range (450–460 nm). In contrast, the sunlight spectrum consists of wavelengths of different colors, roughly in equal proportions. That means that whenever we are exposed to BL from the sun, there is always a strong component of red and near-infrared (NIR) light, which is said to have protective functions. The intensity of BL in screens is 100 times lower compared to sunlight, and also much lower than the doses that could be dangerous (according to a report of SCHEER in 2018). However, it was also criticized that the current officially defined threshold values for phototoxic BL in LED lamps are still too high. In this context, it is possible that usage of some LEDs and monitors is dangerous (especially Gallium Nitride-based (GaN) LEDs) [53].

It is the dose that is dangerous in the end—the cumulative effect of BL exposure could lead to damage in the long term. Therefore, the time spent in front of screens is the main determinant, and current regulations have only been established for acute light exposures; therefore, they do not consider the possible damage of repeated exposures. However, cumulative retinal light exposure, i.e., over a lifetime, is difficult to estimate.

Our data and recent data evaluating BL damage raise concerns about excessive exposure to BL from artificial sources, such as digital screens and specific LEDs containing high amounts of BL, especially in the context of continuous use without interruptions (e.g., staring at screens). Taken together, the role of p53 in light-induced retinal apoptosis may be complex and dependent on the light source, the exposure time, and the wavelengths used, as well as on the investigated cell type and their origin. Depending on the BL and RL content (and specific wavelengths) in an LED, short-time retinal damage may occur due to intense WL exposure. The potential of BL to damage retinal cells may also depend on factors such as age and cumulative exposure over time. While in children and adolescents, large parts of BL can hit the retina and therefore could cause great damage, the clouding of the lens due to aging leads to a diminished BL exposure of the retina. However, as individuals age, the retina’s ability to manage oxidative stress and repair cellular damage also decreases, making the retinal cells more vulnerable to potential damage from BL. Long-term studies on younger generations, who use screens for an increased amount, are needed to determine an accumulative effect of consistently high levels of BL exposure and therefore the impact of BL on late-onset diseases like AMD. Nevertheless, this ex vivo damage model can be used to effectively mimic oxidative-stress-based diseases in a natural way, and therefore also to simulate AMD. This model can help to identify new targets and develop therapeutic options to inhibit retinal cell death induced by oxidative stress. 

## 5. Conclusions

We successfully demonstrated that BL induces oxidative stress and further apoptosis in porcine retinal explants, mimicking AMD pathology. These effects were specific to BL and were not found with single WL exposures. The BL-exposed retinal cultures exhibited exposure-dependent increased p53- and caspase-mediated apoptosis. Inhibition of p53 via pifithrin α caused prevention of retinal cell death. BL may play a role in several ocular diseases, such as AMD, in which an increase of oxidative stress resembles a key mediator. 

## Figures and Tables

**Table 1:** Primary and secondary antibody (AB) dilutions used in immunostainings.

Antibody	Company Primary AB	Dilution Primary AB	Company Secondary AB	Dilution Secondary AB
acetylated (Lys382) p53	Cell Signaling (Danvers, MA, USA)	1:100	Thermo Fisher Scientific (Invitrogen; Karlsruhe, Germany)	1:1000 Alexa Fluor 488
GFAP	BD Bioscience (BP Pharmigen, Heidelberg, Germany)	1:200	Thermo Fisher Scientific (Karlsruhe, Germany)	1:1000 Alexa Fluor 555
Rhodopsin	Abcam (Cambridge, UK)	1:200	Thermo Fisher Scientific (Invitrogen; Karlsruhe, Germany)	1:1000 Alexa Fluor 594

**Table 2:** Primary and secondary antibody dilutions used in Western blots.

Primary Antibody		
Target	Company	Dilution
β-Actin	Cell Signaling, Danvers, MA, USA	1:1000
HSP70	Santa Cruz, Dallas, TX, USA	1:500
NF-kB	Elabscience, Hamburg, Germany	1:500
Rhodopsin	Abcam, Cambridge, UK	1:200
BAX	Cell Signaling, Danvers, MA, USA	1:100
BCL2	Cell Signaling, Danvers, MA, USA	1:100
**Secondary Antibody**		
**Target**	**Company**	**Dilution**
mouse	IRDye^®^ 680RD, LI-COR, Homburg, Germany	1:10,000
rabbit	IRDye^®^ 800CW, LI-COR, Homburg, Germany	1:10,000

**Table 3:** Sequences of primers used for qRT-PCR.

Gene	Sequence 5′-3′	
*Actin-β for*	CACGCCATCCTGCGTCTGGA	XM_003357928.4
*Actin-β rev*	AGCACCGTGTTGGCGTAGAG	
*RPL4 for*	CAAGAGTAACTACAACCTTC	XM_005659862.3
*RPL4 rev*	GAACTCTACGATGAATCTTC	
*GFAP for*	GGAGAAGCCTTTGCTACACG	NM_001244397.1
*GFAP rev*	TCTTCACTCTGCCTGGGTCT	
*Opsin (mws) for*	GGGGAGCATCTTCACCTACA	NM_001011506.1
*Opsin (mws) rev*	GATGATGGTCTCTGCCAGGT	
*Rhodopsin for*	TCCAGGTACATCCCAGAAGG	NM_214221.1
*Rhodopsin rev*	GCTGCCCATAGCAGAAGAAG	
*β-III-tubulin for*	CAGATGTTCGATGCCAAGAA	AK391872.1
*β-III-tubulin rev*	GGGATCCACTCCACGAAGTA	
*TNF-α for*	CCACCAACGTTTTCCTCACT	JF831365.1
*TNF-α rev*	CCAAAATAGACCTGCCCAGA	
*PKCa for*	ACCGAACAACAAGGAACGAC	XM_021066740.1
*PKCa rev*	CTGAGCTCCACGTTTCCTTC	
*HSP70 for*	ATGTCCGCTGCAAGAGAAGT	NM_001123127.1
*HSP70 rev*	GGCGTCAAACACGGTATTCT	
*NF-kB for*	AGGATGGGATCTGCACTGTC	NM_001048232.1
*NF-kB rev*	ATCAGGGTGCACCAAAAGTC	
*BAX for*	AAGCGCATTGGAGATGAACT	XM_003127290.5
*BAX rev*	AAAGTAGAAAAGCGCGACCA	
*Bcl-2 for*	AATTACCATCGGCGTAGTGC	XM_021099593.1
*Bcl-2 rev*	CGTTTCAGCCACCGTAAAAT	

## Data Availability

The data presented in this study are available on request from the corresponding author.

## Supplementary Materials

The following supporting information can be downloaded at: https://www.mdpi.com/article/10.3390/antiox12122072/s1, Figure S1: Blue light induced time and duration dependent cell death in retinal explants, Figure S2: Blue light increased apoptotic cells in exposed whole pig eyes, Figure S3: Inhibition of p53 decreases Müller cell activation after BL-exposure in porcine retinal explants.
