# Supplementary material for: Blue Light Damage and p53: Unravelling the Role of p53 in Oxidative-Stress-Induced Retinal Apoptosis
Source: Antioxidants (Basel). 2023 Dec 4;12(12):2072. doi: 10.3390/antiox12122072 (PMC10740515; doi:10.3390/antiox12122072)
Supplement: Supplementary file 1 [file antioxidants-12-02072-s001.zip › antioxidants-2685571-supplementary.pdf]

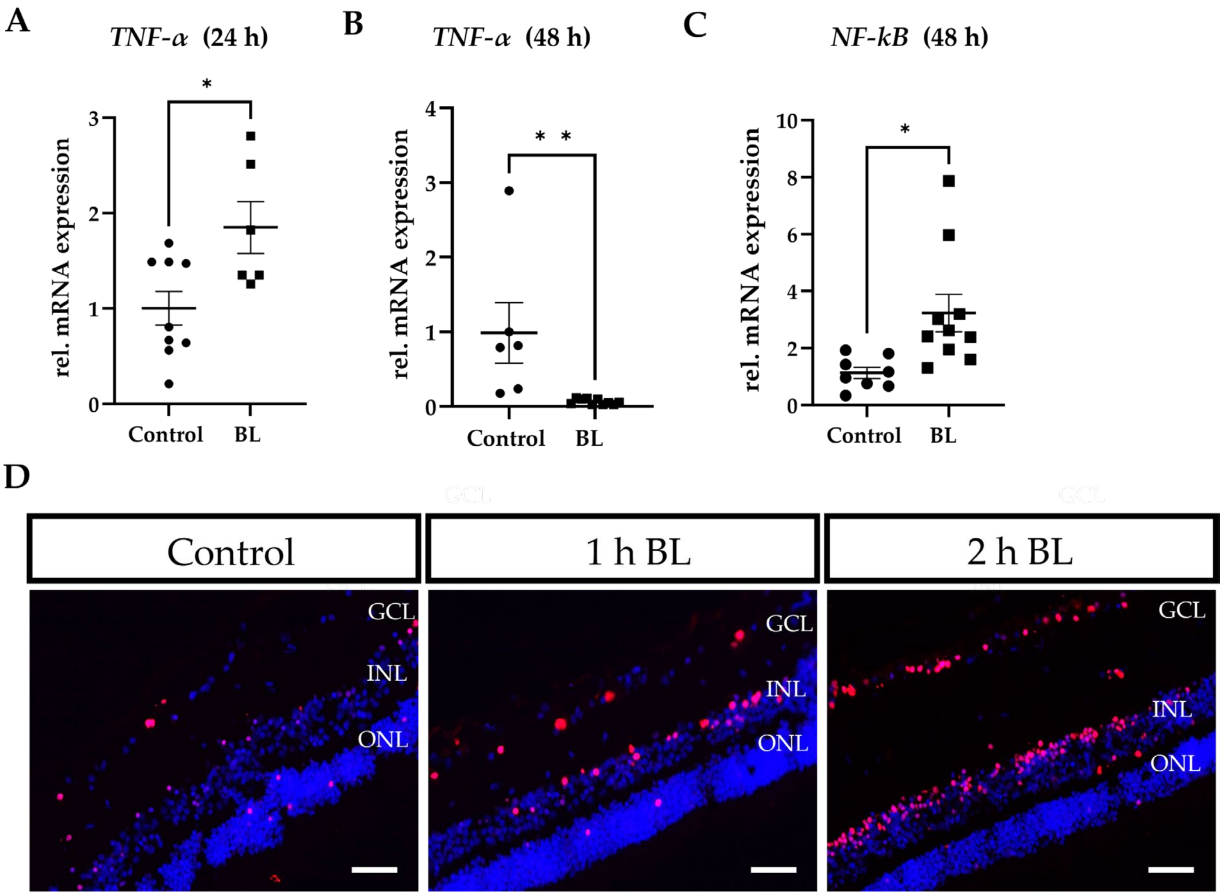

Figure S1. Blue light induced time and duration dependent cell death in retinal explants.

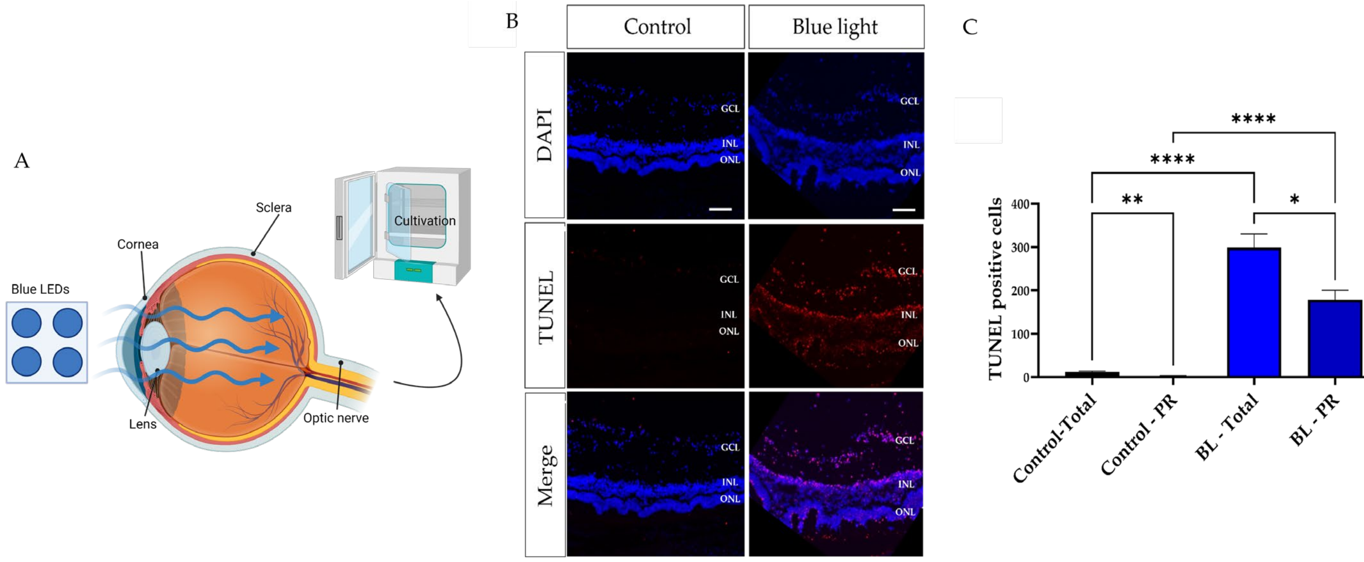

Figure S2. Blue light increased apoptotic cells in exposed whole pig eyes.

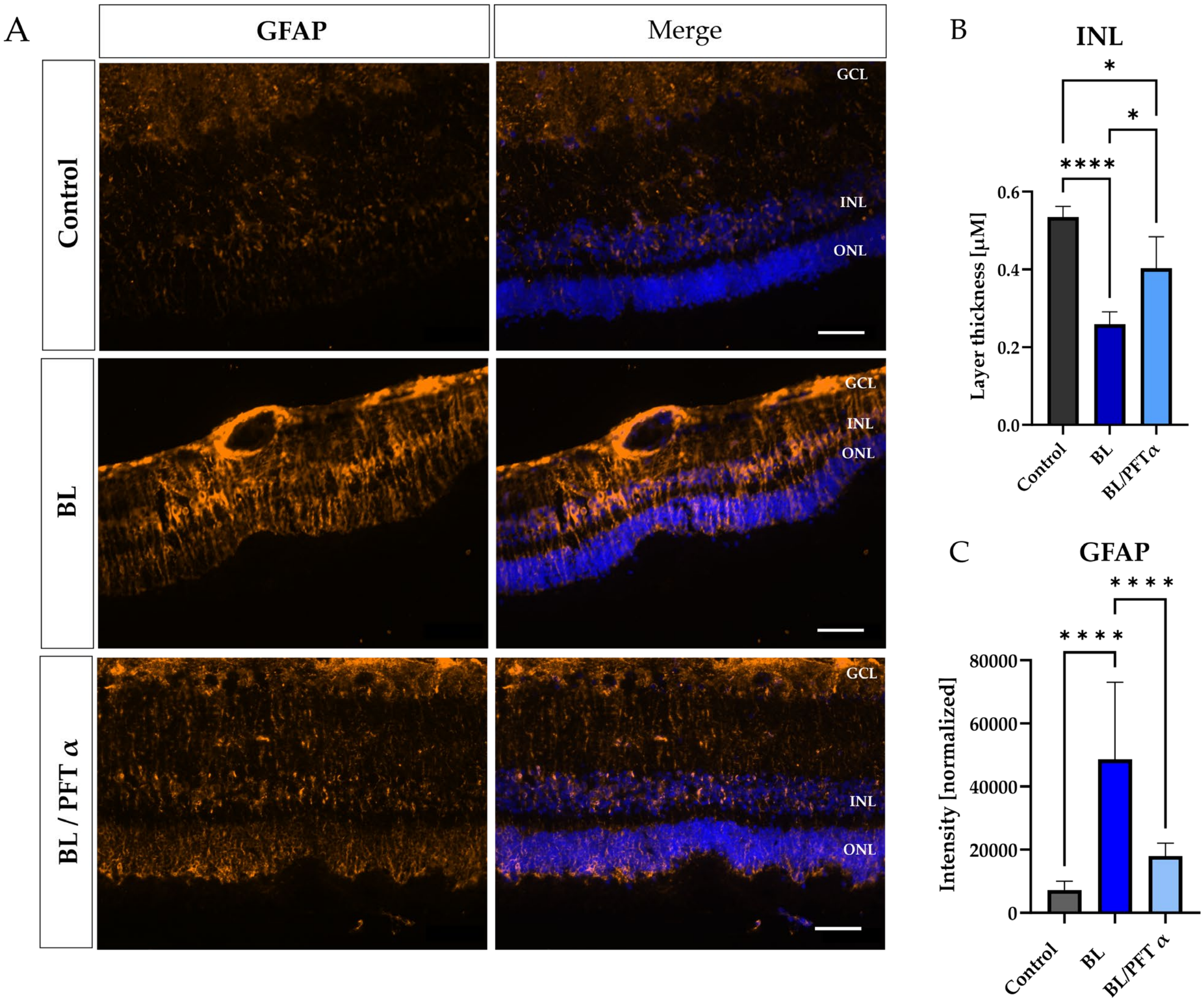

Figure S3. Inhibition of p53 decreases Müller cell activation after BL-exposure in porcine retinal explants.
